# Tocilizumab treatment safety in rheumatoid arthritis in a patient with multiple sclerosis: a case report

**DOI:** 10.1186/1756-0500-7-641

**Published:** 2014-09-12

**Authors:** Hiroe Sato, Daisuke Kobayashi, Asami Abe, Satoshi Ito, Hajime Ishikawa, Kiyoshi Nakazono, Akira Murasawa, Takeshi Kuroda, Masaaki Nakano, Ichiei Narita

**Affiliations:** Department of Rheumatology, Niigata Rheumatic Center, 1-2-8 Honcho, Shibata City, Niigata, 957-0054 Japan; Division of Clinical Nephrology and Rheumatology, Niigata University Graduate School of Medical and Dental Sciences, 1-757 Asahimachi-Dori, Chuoku, Niigata City, Niigata, 951-8510 Japan; Department of Medical Technology, School of Health Sciences, Faculty of Medicine, Niigata University, 2-746 Asahimachi-Dori, Chuoku, Niigata City, 951-8518 Japan

**Keywords:** Rheumatoid arthritis, Multiple sclerosis, Tocilizumab, Interleukin-6, Tumour necrosis factor

## Abstract

**Background:**

Multiple sclerosis is a relatively rare disease, and complications of multiple sclerosis and rheumatoid arthritis are much rarer. Since anti-tumor necrosis factor therapy increases exacerbations of multiple sclerosis, complications of demyelinating diseases contraindicate anti-tumor necrosis factor therapy. There have been few reports of anti-interleukin-6 receptor therapy for patients with rheumatoid arthritis complicated with multiple sclerosis.

**Case presentation:**

A 53-year-old Japanese woman with multiple sclerosis and rheumatoid arthritis was admitted to our hospital because her rheumatoid arthritis was uncontrolled with oral methotrexate, tacrolimus, and prednisolone. She had developed multiple sclerosis when she was 25 years old and was treated with glucocorticoid therapy. Her multiple sclerosis was in remission for more than 9 years. Because anti-tumour necrosis factor therapy can exacerbate demyelinating disease, the anti-interleukin-6 receptor antibody tocilizumab was started at 8 mg/kg every 4 weeks. At the second administration of tocilizumab, complete remission was achieved. She has remained in remission with tocilizumab without recurrence of multiple sclerosis for more than 5 years.

**Conclusion:**

Anti-interleukin-6 therapy was safely used in this patient with rheumatoid arthritis without exacerbations of multiple sclerosis.

## Background

Multiple sclerosis (MS) is an autoimmune, inflammatory, demyelinating disease of the central nervous system characterised by repeated relapses and remissions. The prevalence rate of MS in Japan is reportedly 8 to 9 per 100,000 persons. High-dose glucocorticoid therapy is used for initial and relapsed progression of MS with or without immunosuppressive agents. Although MS is an autoimmune inflammatory disease, as is rheumatoid arthritis (RA), and although the level of tumour necrosis factor (TNF) in cerebrospinal fluid is correlated with the severity and progression of the disease [1], anti-TNF therapy fails and actually increases exacerbations [2, 3]. Therefore, complications of demyelinating diseases contraindicate anti-TNF therapy. There have been few reports of anti-interleukin (IL)-6 receptor therapy for patients with RA complicated with MS.

We herein describe a patient with RA and MS treated with anti-IL-6 receptor therapy.

## Case presentation

A 53-year-old Japanese woman was admitted to Niigata Rheumatic Centre, Shibata city, Japan. She had been diagnosed with MS associated with right optic neuritis and thoracic myelitis when she was 25 years old and treated with high-dose prednisolone (PSL). The myelitis had relapsed three times when she was 36, 37 and 40 years old and treated with high-dose PSL. Oligoclonal IgG band was found in cerebral spinal fluid (CSF) and IgG and myelin basic protein in CSF were elevated (4.9 mg/dL and 1.2 mg/dL, respectively). Brain T2 weighted magnetic resonance imaging (MRI) showed high intensity area beside left lateral ventricle indicating asymptomatic plaque lesion due to MS. High intensity area was also shown in T2 weighted MRI of cervical spinal cord. Anti-aquaporin 4 antibody was negative. Slight right hemiparesis remained, and she needed a cane to walk outside. The MS achieved remission and PSL was stopped for 9 years. When she was 50 years old, polyarthritis developed, and rheumatoid factor and C-reactive protein (CRP) levels were high. She was diagnosed with RA. The PSL was restarted at 7.5 mg daily and methotrexate (MTX) was begun. Because the MTX could not be increased over 8 mg/week because of mild elevation of transaminases, tacrolimus (3 mg daily; TAC) was added to MTX and leukocyte apheresis was performed. However, the RA activity remained high: the CRP was 2.3 mg/dL and the disease activity score (DAS28ESR) was 4.94 (moderate disease activity). Furthermore, joint space narrowing of both knees and ankles had progressed obviously over 1 year. Because anti-TNF therapy can exacerbate demyelinating disease, the anti-IL-6 receptor antibody tocilizumab (TCZ) was started at 8 mg/kg every 4 weeks. At the second administration of TCZ, the CRP was <0.1 mg/dL and the DAS28ESR was 2.0 (complete remission). The MTX and TAC were tapered and stopped in 6 months, and the PSL was tapered to 0.5 mg daily in 1 year. The health assessment questionnaire disability index (HAQ DI) in 1 year was 1.88 and functional disability was remained. At the 5-year follow-up, she remained in remission with TCZ.

Serum interferon (IFN) -γ was negative (≤0.1 IU/mL) and serum high sensitivity TNF-α was within normal range (1.6 pg/mL) before starting TCZ therapy. Both of them kept the same levels for a year. Serum IL-6 level was elevated, 51.2 pg/mL (normal range; ≤4.0 pg/mL) before starting TCZ therapy and it was 57.1 pg/mL a year later.

## Discussion

Complications of MS and RA are rare, and only a few cases have been reported [4, 5]. In two case reports, the duration between the onset of each disease was long (8–20 years), and RA or MS was preceded by the other and the two diseases did not flare at the same time [4, 5]. Our patient with MS also developed RA 25 years after the onset of MS, which was well controlled without medication for 9 years. Thus, the onset mechanisms of MS and RA are expected to fundamentally differ despite the fact that both are autoimmune inflammatory diseases.

On the other hand, there are several reports of patients with RA who developed demyelinating diseases during anti-TNF therapy [6–8] and patients with MS who developed inflammatory arthritis during IFN-β therapy [9, 10]. The mechanisms of demyelinating disease induced by anti-TNF therapy are not clear, but inhibition of TNF leads to IFN-γ production, which is associated with MS [11]. Moreover, TNF polymorphism may be associated with anti-TNF therapy-induced demyelinating diseases [12]. IFN-β therapy for preventing recurrence of relapsing-remitting MS and secondary progressive MS is now widely used. Arthritis reportedly develops during IFN-β therapy in patients with MS, and the HLA phenotype may be involved in its pathogenesis [9]. Elevated levels of IL-6 in the serum in response to IFN-β therapy may also be associated with arthralgia [10].

In comparison, few studies have reported anti-IL-6 therapy in demyelinating disorders. A case report of a 72-year-old woman with leukoencephalopathy that developed in a Phase 3 clinical trial of TCZ for treating RA has been published [13]. However, the relationship between TCZ and leukoencephalopathy was not clear because the possibility of infection had not been completely excluded and the symptoms did not improve after discontinuing TCZ.

Studies involving mouse models of MS (experimental autoimmune encephalomyelitis, EAE) and RA (collagen-induced arthritis, CIA) have revealed that Th-17 plays an important role in the development of both diseases [14, 15]. Furthermore, IL-6 mainly affects the differentiation of Th-17 in the mouse, and anti-IL-6 therapy inhibits the onset of EAE and CIA. Meanwhile, TNF affects local inflammation [14, 15]. Thus, anti-IL-6 therapy may control EAE and CIA earlier and more radically than anti-TNF therapy. In humans, IL-6 mainly affects the differentiation of Th-17, as in the mouse, and anti-IL-6 therapy should theoretically inhibit both RA and MS. However, the cytokine pathways differ in humans and mice, and further studies are needed to determine whether anti-IL-6 therapy can be used safely in patients with MS.

## Conclusion

Here, we reported a patient with RA complicated by MS who was treated with anti-IL-6 therapy for more than 5 years without an exacerbation of the MS. Because anti-TNF therapy can induce and worsen demyelinating diseases, anti-IL-6 therapy is a potential treatment for patients with RA complicated by MS.

## Consent

Written informed consent was obtained from the patient for publication of this Case Report. A copy of the written consent is available for review by the Editor-in-Chief of this journal.

## Abbreviations

RA: Rheumatoid arthritis
MS: Multiple sclerosis
TNF: Tumour necrosis factor
IL: Interleukin
PSL: Prednisolone
CSF: Cerebral spinal fluid
MRI: Magnetic resonance imaging
CRP: C-reactive protein
MTX: Methotrexate
TAC: Tacrolimus
TCZ: Tocilizumab
IFN: Interferon
EAE: Experimental autoimmune encephalomyelitis
CIA: Collagen-induced arthritis.
